# Assessment of Repetitive Controlled Ovarian Stimulation (COS) Cycles on Oocyte Donors: Impact on Oocyte Quality and Viable Embryo Yield

**DOI:** 10.1007/s43032-024-01584-x

**Published:** 2024-05-20

**Authors:** Zalihe Yarkiner, Fazilet Kübra Boynukalın, Önder Coban

**Affiliations:** 1 Department of Basic Sciences and Humanities, Faculty of Arts and Sciences, Cyprus International University, Nicosia, Cyprus; 2 Infertility Department, Turkey, Obstetrics and Gynaecology, Istanbul, Bahçeci Fulya IVF Center, Üsküdar University, Istanbul, Turkey; 3 British Cyprus IVF Hospital, Embryology, Nicosia, Cyprus

**Keywords:** IVF, Egg donation, Egg donor, Repeated COS, Embryo quality

## Abstract

The utilization of donor eggs has broadened the options for Assisted Reproductive Technology (ART) among women facing challenges with egg quantity or quality. Given that donors are typically selected from young and fertile individuals, In Vitro Fertilization with egg donation (IVF-ED) tends to exhibit higher rates of implantation, pregnancy, and live births compared to IVF with the woman's own eggs, especially for females over 35 years old. This has led to a projected increase in the demand for IVF-ED, surpassing the number of available donors. Consequently, many centers opt to use oocyte donors for multiple cycles. However, the correlation between repeated Controlled Ovarian Stimulation (COS) cycles and the performance of donors in terms of viable blastocyst stage embryo (VEC) or blastocyst embryo rate is not definitively established and remains of interest. This study aims to explore the preimplantation characteristics of embryo development and oocyte maturation status based on the number of donor COS cycles, employing a Generalized Linear Mixed Model (GLMM) framework. The study encompasses 1965 embryo transfer (ET) cycles involving 399 donors who underwent a minimum of two and a maximum of nine controlled ovarian hyperstimulation (COS) cycles. The findings indicate that, with the patient undergoing six or more cycles of ovarian stimulation, despite a 3.9% increase in both maturation and fertilization rates, there is a corresponding decrease of 4.5% in VEC rate and 4.7% in blastulation rates. In essence, an escalating number of donor COS cycles appears to be associated with a disadvantageous reduction in embryo quality.

## Introduction

Oocyte donation has emerged as a prominent treatment option for infertility, particularly among women aged 40 and above, where assisted reproductive technology (ART) interventions are frequently employed. This approach extends to cases involving premature ovarian failure, diminished ovarian function, recurrent pregnancy loss, maternal genetic abnormalities, and unsuccessful in vitro fertilization (IVF) attempts. The utilization of donor eggs has become crucial in addressing challenges related to insufficient egg quantity or quality, reflecting a significant portion of ART cycles globally, such as 22% in Latin America [1], approximately 10% in the USA [2], and a comparable percentage in Europe [3]. The increasing reliance on ART over the past decade, with more than a doubling of usage in the USA [2], underscores its importance in addressing infertility.

With the doubling of ART usage in the USA over the past decade, this innovative approach has become crucial in addressing a spectrum of infertility challenges. ART interventions play a particularly essential role for individuals facing obstacles such as premature ovarian failure, diminished ovarian function, recurrent pregnancy loss, maternal genetic abnormalities, and unsuccessful IVF attempts. The utilization of donor eggs through oocyte donation is pivotal in overcoming obstacles related to insufficient egg quantity or quality, making it a vital component of ART cycles worldwide.

Despite the success rates associated with in vitro fertilization using oocyte donation (IVF-OD), which demonstrates better implantation, pregnancy, and live birth rates than IVF with autologous oocytes in women over 35 years [4], a critical issue persists. The increasing demand for IVF-OD is predicted to surpass the number of available donors, prompting many centers to utilize oocyte donors for multiple cycles [5]. However, the absence of a standardized protocol regarding the upper limit of oocyte donation cycles reveals a fundamental gap in understanding the unambiguous ramifications of repeated COS cycles on the embryogenic efficacy exhibited by oocyte donors. To address this concern, the American Society for Reproductive Medicine (ASRM), a prominent authority in reproductive health, tentatively suggested in their 2020 committee opinion limiting donor cycles to no more than six [6], highlighting the need for further research to establish definitive recommendations.

Gougeon's hypothesis posits that follicle development from the early to the preovulatory stage spans approximately 80 to 90 days, with gonadotropin influence becoming significant only in the final two weeks. Furthermore, in assisted reproductive technology, controlled ovarian stimulation (COS) doesn't necessarily result in a greater number of follicles than those naturally selected by the ovary. Instead, repeated COS with higher gonadotropin doses appears to offer a mechanism through which recruited ovarian follicles evade regression [7, 8]. Additionally, in mice, it was found that repeated ovarian stimulation led to oxidative stress in the ovaries and an extension of the luteal phase, during which the corpus luteum forms [9, 10].

After delving into Gougeon's hypothesis on follicle development and COS, our focus shifts to the tangible physiological impacts of repeated COS cycles. This exploration unravels how hormonal stimulations may disrupt the delicate balance within the donor's reproductive system, potentially affecting follicular development, oocyte maturation, and subsequent embryo quality. Moreover, the cumulative effects of repeated COS may extend beyond the immediate cycle, influencing endometrial receptivity and impacting implantation and pregnancy outcomes.

Understanding these intricate physiological pathways is crucial for assessing the potential risks associated with multiple oocyte donation cycles. The prolonged exposure to ovarian stimulation protocols may contribute to conditions such as ovarian hyperstimulation syndrome (OHSS) and pose risks to the cardiovascular and metabolic health of donors. This comprehensive insight is paramount for formulating evidence-based guidelines that ensure the well-being of oocyte donors.

In addressing the intricate physiological impacts of repeated COS cycles, our research employs Generalized Linear Mixed Models (GLMMs) as a sophisticated analytical tool. This choice is deliberate and aligns seamlessly with our study's primary objectives. GLMMs offer a nuanced approach to analyze complex data structures, making them particularly suited to capture the dynamic nature of physiological responses across multiple cycles and donors.

The utilization of GLMMs is pivotal in our quest to explore not only the average impact but also individual variations in response to multiple oocyte donation cycles. By incorporating random effects, GLMMs enable us to model the inherent heterogeneity within the data, providing a more comprehensive understanding of the longitudinal implications of repeated COS regimens. This analytical approach is well-suited for unraveling the complexities of our dataset, which involves repeated measures, longitudinal data, and clustered observations.

In essence, the application of GLMMs represents a strategic choice to enhance the depth and robustness of our analysis. Through this methodological approach, we aim to contribute novel insights into the physiological consequences of iterative COS regimens, ultimately advancing our understanding and informing evidence-based guidelines for oocyte donation.

In conclusion, our study delves into the intricate physiological pathways influenced by repeated COS cycles in oocyte donation. Utilizing GLMMs, we aim to uncover nuanced insights into these dynamic impacts, ultimately paving the way for evidence-based guidelines. By contributing novel perspectives, our research seeks to advance understanding in ART, prioritizing the well-being of donors and ushering in a new era of precision and care.

## Materials and Methods

In this study, retrospective analysis of donors undergoing multiple ICSI cycles at the British Cyprus IVF center between 2010 and 2019 was conducted with the aim of assessing the effect of repeated COS cycles on ovarian response, which was determined using the VEC and blastulation rates of the donors per cycle, as the number of cycles varied. We used the data of cycles with only fresh oocytes due to the possible effect on the results depending on freezing and thawing the oocytes. This study included a total of 1965 embryo transfer (ET) cycles comprising 399 donors who underwent a minimum of two and a maximum of nine controlled ovarian stimulation (COS) cycles. Inclusion criteria were the cycles with fresh oocytes, ejaculated sperm, donor ages below 35 years and fulfillment of donor screening parameters explained in the following section. The study was assessed and approved by the Institutional Review Board (BIVF-IRB-9).

### Donor Screening

Donors aged 19–35 years with no family history of congenital malformations or hereditary diseases were considered; all donors were tested for hepatitis B surface antigen, hepatitis C virus, human immunodeficiency virus antibodies, syphilis, thalassemia, and cystic fibrosis. All donors had a normal karyotype and normal gynecological examinations, without any endocrine abnormalities. All donors were informed about the risks associated with COS cycles and had signed informed consent forms.

### Ovarian Stimulation Protocol

COS was performed using the GnRH antagonist protocol. Recombinant FSH (150–300 IU, Gonal-F; Serono) and/or hMG (75 − 150 IU; Menogon; Ferring) was administered on day 2 of the menstrual period. Starting on the sixth day of COS, ovarian response was monitored via serial transvaginal ultrasound (TVUSG) and serum estradiol and progesterone levels. When the leading follicle exceeded 13 mm in diameter, 0.25 mg of GnRH antagonist (Cetrotide; Serono) was started daily until the day of the last trigger. Triptorelin (Gonapeptyl, Ferring) 200 µg was administered when at least two follicles reached 18 mm in diameter. Oocyte retrieval was performed 34–36 h after the last trigger under ultrasound guidance.

### Embryo Morphology Assessment

The ICSI method was used for fertilization of the oocytes. Fertilization was assessed as described in the ESHRE Guideline; the presence of two pronuclei with two polar bodies was considered normal fertilization [7]. Day 3 embryos were graded as described by Ciray et al. (2012), where the number of cells, nuclear visibility, number of nuclei, blastomere uniformity, degree of fragmentation, and cytoplasmic characteristics were recorded [8]. Blastocyst grading was performed as described by Gardner and Schoolcraft [10]. The VEC was calculated as the total number of embryos at the grades greater or equal to 3BB on day 5 and 6 of embryo development divided by the number of fertilized oocytes. Similarly, blastocyst embryo count (BEC) was calculated as the total number of embryos graded above early blastocyst (1 or 2) divided by the number of fertilized oocytes.

### Analyzed Variables

The total number of oocytes used, total metaphase II oocytes (MII) used, maturation rate, fertilization rate, number of frozen embryos, VEC rate, and blastulation rate, which were all measured per donor per cycle, and the donor’s age were analyzed.

### Sample Size

To calculate the sample size required for this study, R software program version 4.1.1 was used with the *longpower* package version 1.0.23. To fulfill the aim of the study each of the dependent parameters, including total number of oocytes used, total MII used, maturation rate, fertilization rate, number of frozen embryos, VEC rate, and blastulation rate, which were all measured per donor per cycle. Thereafter, the aim was to produce a generalized linear mixed effect model (GLMM) based on the VEC rate and blastulation rate per donor per cycle using a multilevel modeling structure where a minimum of two and maximum of nine cycles per donor was used. Therefore, the *lmmpower* command of sample size calculation for longitudinal data was used, taking the alpha level as 5%, power of the study as 80%, the change in the pilot estimate of VEC rate and blastulation rate as 0.01, the pilot estimate of random intercept as 0.001, the pilot estimate of residual variance as 0.021, and the pilot estimate of variance of random slope as 0.001, considering the correlation between VEC rate and number of COS cycles and the correlation between blastulation rate and COS cycles as 0.006. The results of the power analysis suggested that 374 donors were required to be 80% certain that one limit of a two-sided 95% confidence interval would exclude a difference of more than 0.01. In addition, if a 6.7% missing data rate is considered, an extra 25 patients should be included, which increases the total sample size to 399 donors.

### Study Design

Each donor underwent multiple repeated COS cycles (Timeframes between stimulation protocols varies between 4 to 8 months) with a minimum of two and maximum of nine cycles per donor, resulting in a total of 1965 ET transfers. Therefore, we had a two-level model structure where we had repeated COS cycles for each donor. The donors were first grouped into six categories based on the number of COS cycles. Donors with cycle 1 (i.e., a first cycle) were included in the first group (n = 399). Donors with cycle 2 in addition to cycle 1 (i.e., two completed cycles) were included in the second group (n = 399). Donors who completed three cycles were included in the third group (n = 279), donors who completed four cycles were included in the fourth group (n = 223), donors who completed five cycles were included in the fifth group (n = 178), and donors who completed six, seven, eight, or nine cycles were included in a sixth group (n = 153), where 92/153 donors completed nine cycles.

### Statistical Analysis

To determine the effect of repeated COS cycles on the total number of oocytes used, total MII used, maturation rate, fertilization rate, number of frozen embryos, VEC rate, and blastulation rate, which were all measured per donor per cycle, SPSS 25.0 software was used for data analysis. All the mentioned variables were continuous variables. Therefore, normality tests, namely the Kolmogorov–Smirnov and Shapiro–Wilk tests, were performed on these variables and concluded that the data were non-normally distributed. The median values of these data with the corresponding minimum and maximum values are reported in Table 1, and the independent median test was used to evaluate whether there was a significant difference in median values of such parameters between different categories of COS cycles. Spearman’s correlation coefficient was used to analyze the correlation between COS cycle number and each of these parameters, where the r value indicates Spearman’s correlation coefficient.

**Table 1:** Statistics per OPU

	I. OPU	II. OPU	III. OPU	IV. OPU	V. OPU	VI-IX OPU	*p*-value
*Number of oocytes retrieved*	30 (0–99)	32 (0–96)	35 (6–100)	35 (6–90)	38 (11–106)	39 (8–105)	< 0.0001
Number of M2 used*	13.50 (2–26)	14 (4–25)	14 (6.33–25)	14 (5–27)	14 (6–22)	13.67 (6–22.50)	0.4
*Fertilization Rate*	75 (0–100)	75 (20–100)	73.33 (10–100)	75 (28–100)	75.30 (13–100)	76.19 (20–100)	0.053
*Maturation Rate*	80 (3–100)	80 (3–100)	79.31 (11–100)	80.77 (13–100)	82.32 (3–100)	83.33 (3–100)	< 0.0001
*VEC Rate*	56 (0–100)	50 (5–100)	51.19 (0–100)	52.33 (9–100)	52.63 (0–100)	50 (0–100)	0.559
*Blast Rate*	57 (0–100)	54.29 (0–100)	55.56 (0–100)	53.94 (9–100)	54.55 (0–100)	50 (0–100)	0.606
*Number of Transferred embryos*	2.75 (0–4)	2.50 (0–5)	2.33 (0–3.67)	2.33 (0–4)	2.50 (0–4)	2.33 (0–4)	< 0.0001
*Total M2*	23 (0–80)	25 (0–83)	27 (3–82)	28 (4–82)	30 (0–96)	32 (0–88)	< 0.0001
*Pregnancy*	65.8%	64.6%	64.4%	68.2%	73.2%	69.1%	0.496
*Live Birth*	52%	50.3%	48.2%	51%	57.7%	55%	0.519

^*^The number of mature oocytes (M2) used for individual recipient cycle

Further analysis of this data included modeling of each of the parameters (total number of oocytes used, total MII used, maturation rate, fertilization rate, number of frozen embryos, VEC rate, and blastocyst embryo count, which were all measured per donor per cycle) as a dependent variable to determine the effect of COS cycle number, taking COS cycle group as the only independent variable at cycle level. In this model, because the data had a two-level structure, GLMMs were used to consider both the fixed effect of the COS cycle group and the random effect of the donor. In producing GLMM models, due to the large number of cycles (1965 cycles), the data were assumed to follow a normal distribution, and identity link function was used. Furthermore, to make inferences on donor performance, VEC rate and blastulation rate were used as dependent variables to produce models on donor performance, taking into account the two-level structure of the data, accounting for fixed and random effects using GLMM models, and investigating the effect of repeated COS cycles by controlling for donor age, maturation rate, and fertilization rate. P-values < 0.05 were considered statistically significant.

## Results

### Basic Characteristics of the Patients

#### General Description of the Donor Population

In total, data from 399 donors were used in this study. Each donor underwent a minimum of two and a maximum of nine cycles. The overall mean age of the donors was 25.45 ± 4.074 years, ranging from 19 to 35 years, and there were 102 donors (25.56%) below the age of 23 years. A total of 1965 ETs were performed among the 399 donors.

### Independent Median Test to Compare Laboratory Indicators and Pregnancy Outcomes Between COS Cycle Groups

**Fig. 1:**
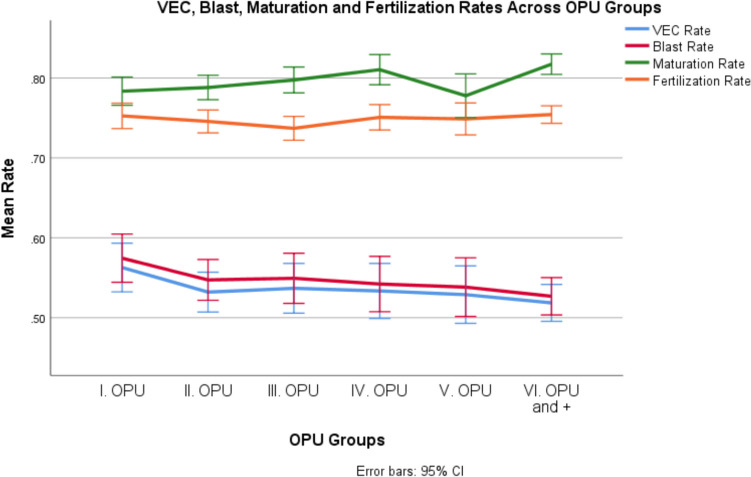
Maturation, Fertilization Rates, VEC and Blastocyst Rates Across OPU Groups. VEC: Number of blastocytes at grades equal or grater than 3BB. Blast Rate: Number of blastocytes at grades equal or grater than early blastocytes (grades 1 and 2). Maturation rate: Number of mature oocytes/total number of oocytes collected. Fertilization rate: Number of fertilized oocytes (2PN and 2polar body)/number of mature oocytes injected (M2)

The results presented in Table 1 show that the median maturation rate per donor per cycle (80, 80, 79.31, 80.77, 82.32, 83.33 for cycle group 1, 2, 3, 4, 5 and 6 respectively; p < 0.0001), total number of oocytes used per donor per cycle (30, 32, 35, 35, 38, 39 for cycle group 1, 2, 3, 4, 5 and 6 respectively; p < 0.0001), and total MII used per donor per cycle (23, 25, 27, 28, 30, 32 for cycle group 1, 2, 3, 4, 5 and 6 respectively; p < 0.0001) were significantly different between the COS cycle groups. The comparative analysis of mean rates pertaining to Maturation, Fertilization, VEC, and Blastocyst outcomes across OPU groups is additionally presented in Fig. 1.

When pairwise comparison was performed on maturation rate per donor per cycle to determine which COS cycle groups were responsible for the significant difference, we found that cycle group 6 was significantly different compared with cycle group 2 (p < 0.0001) and cycle group 3 (p = 0.005). Furthermore, the maturation rate per donor per cycle was higher in cycle group 6 (83.33%) than in both cycle group 2 (80%) and cycle group 3 (79.31%).

Regarding the total number of oocytes used per donor per cycle, compared with cycle group 1, significant differences were observed in cycle group 3 (p = 0.025), 4 (p = 0.001), 5 (p < 0.0001), and 6 (p < 0.0001). The total number of oocytes used per donor per cycle was lowest in cycle group 1 (30) compared with the other cycle groups (35 in cycle groups 3 and 4, 38 in cycle group 5, and 39 in cycle group 6). In addition, significant differences regarding the total number of oocytes used were observed between cycle groups 2 and 5 (32 [0–96] and 38 [11–106], respectively; p = 0.041), 2 and 6 (32 [0–96] and 39 [8–105], respectively; p < 0.0001), and 3 and 6 (35 [6–100] and 39 [8–105], respectively; p = 0.010). Therefore, as the number of repeated COS cycles increased, the total number of oocytes used per donor per cycle also increased significantly.

Regarding the total MII used per donor per cycle, compared with cycle group 1, significant differences were observed in cycle group 3 (p = 0.021), 4 (p = 0.014), 5 (p = 0.001), and 6 (p < 0.0001). The total MII used per donor per cycle was lowest in cycle group 1 (23) compared with the other groups (27, 28, 30, and 32 in cycle groups 3, 4, 5, and 6, respectively). In addition, significant differences regarding the total MII used were observed between cycle groups 2 and 5 (25 [0–83] and 30 [0–96], respectively; p = 0.020), 2 and 6 (25 [0–83] and 32 [0–88], respectively; p < 0.0001), and 3 and 6 (27 [8–32] and 32 [0–88], respectively; p = 0.003). Therefore, as the number of repeated COS cycles increased, the total number of MII used per donor per cycle also increased significantly.

Moreover, reproductive outcomes were examined concerning repeated donor COS cycles, revealing no statistically significant differences in relation to both pregnancy and live birth (Table 1).

Prior to employing the GLMM, Spearman's correlation coefficient analysis was conducted to elucidate the relationships between OPU Groups and donor age. Additionally, correlations were explored between OPU Groups and average values calculated at the donor level for several factors initially measured at the cycle level. These factors included fertilization rate, number of 2PN, total number of thawed embryos, total number of oocytes used, total M2 used, number of embryos transferred, number of embryos frozen, VEC rate, and the number of blastocyst embryos.

Furthermore, correlations were investigated between OPU Groups and average values calculated at the donor level for factors initially measured at the OPU level, such as the total number of oocytes, total M2, Abortus rate, and Maturation rate. These analyses were crucial for comprehensively understanding the interplay between OPU Groups and various reproductive parameters, shedding light on the nuanced effects of repeated COS cycles within the studied population.

The Spearman's correlation coefficient test revealed a non-significant correlation between donor age and OPU groups (p = 0.054). However, other parameters demonstrated statistically significant relationships with OPU groups at the donor level.

The average value of 2PN embryos at the donor level had a subtle but statistically significant positive correlation (r = 0.045, p = 0.048) with OPU groups. Similarly, the average value of thawed embryos (r = 0.113, p =  < 0.0001) and average number of frozen embryos (r = 0.062, p = 0.007) all exhibited positive correlations with OPU groups at the donor level.

Additionally, the average number of total oocytes (r = 0.155, p =  < 0.0001), average number of M2 (metaphase II) (r = 0.160, p =  < 0.0001), and average number of abortus rate (r = 0.074, p = 0.001) showed statistically significant positive correlations with OPU groups at the donor level.

In the realm of reproductive sciences, these findings highlight the intricate relationships between repeated OPU cycles and various reproductive parameters, offering insights into embryonic development, cryopreservation outcomes, and oocyte quantity.

### Analyzing Effect of Repeated COS cycles on Each Dependent Variable Using GLMM Models

To assess donor performance, four separate models were initially computed using GLMM models, taking the dependent variable as i) maturation rate per donor per cycle, ii) fertilization rate per donor per cycle, iii) VEC rate per donor per cycle, and iv) blastulation rate per donor per cycle. The independent variable was the COS cycle group. In all four models, the reference group used in the independent variable COS cycle groups was cycle group 1, that is, the effect of a patient having repeated COS cycles on the outcome variable was compared to cycle group 1.

From the results of the GLMM models for maturation rate, fertilization rate, VEC rate, and blastulation rate per donor per cycle, the donors who underwent 2, 3, 4, or 5 COS cycles did not have significantly different maturation rates, with a mean rate of 77% (p = 0.520, p = 0.064, p = 0.055, and p = 0.904 for cycles 2, 3, 4, and 5, respectively, compared to cycle 1), nor did they have significantly different fertilization rates, with a mean rate of 72.1% (p = 0.154, p = 0.682, p = 0.142, and p = 0.283 for cycles 2, 3, 4, and 5, respectively, compared to cycle 1), VEC rates, with a mean rate of 56.3% (p = 0.140, p = 0.240, p = 0.209, and p = 0.155 for cycles 2, 3, 4, and 5, respectively, compared to cycle 1), or blastulation rates, with a mean rate of 57.5% per donor per cycle (p = 0.194, p = 0.254, p = 0.165, and p = 0.132 for cycles 2, 3, 4, and 5, respectively, compared to cycle 1). Significant differences in maturation rate (p < 0.0001), fertilization rate (p = 0.020), VEC rate (p = 0.021), and blastulation rate (p = 0.015) were observed only for donors who underwent six or more COS cycles compared to one cycle. Therefore, ovarian response measured in terms of VEC rate and blastulation rate was similar in patients undergoing two to five cycles of ovarian stimulation. However, when the patient underwent six or more cycles of ovarian stimulation, even when both the maturation rate per donor per cycle and fertilization rate per donor per cycle increased by 3.9% and 2.2%, respectively, the VEC rate per donor per cycle and blastulation rate per donor per cycle decreased by 4.5% and 4.7%, respectively.

To make further conclusions on donor performance, two additional GLMM models were computed using i) the VEC rate per donor per cycle (Table 2) and ii) the blastulation rate per donor per cycle (Table 3) as dependent variables. To identify the factors affecting the VEC and blastulation rates per donor per cycle, fertilization rate and maturation rate per donor per cycle were first grouped into two categories each. If the donor’s fertilization rate per cycle was below the average rate, the donor was assigned to group 1; if the donor’s fertilization rate per cycle was above the average rate, the donor was assigned to group 2. A similar process was performed for maturation rate per donor per cycle, which was treated as a categorical variable in the model. This enabled us to determine whether the VEC and blastulation rates per cycle were affected by the donor having below or above average rates of fertilization and maturation per cycle. Therefore, in such models and in COS cycle groups, donor age, fertilization rate, and maturation rate per donor per cycle, which were treated as categorical variables, were added to the model as independent variables. The analytical results derived from the two models suggest that factors such as donor age, fertilization rate, and maturation rate exhibit statistically non-significant associations with the VEC rate (p = 0.161, p = 0.439, p = 0.057, respectively) and the blastulation rate per donor per cycle (p = 0.805, p = 0.271, p = 0.219, respectively). Upon meticulous adjustment for the covariates of donor age, fertilization rate, and maturation rate per donor per cycle, our investigation demonstrates a noticeable attenuation in donor performance, as measured by the VEC rate per cycle (p = 0.034) and the blastulation rate per cycle (p = 0.024), as IVF cycles extend beyond the fifth cycle. In specific terms, the VEC rate per cycle exhibits a decrease of 4.2%, while the blastulation rate per cycle witnesses a reduction of 4.5%. These findings underscore the significance of accounting for the cumulative effect of IVF cycles on donor performance metrics.

**Table 2:** GLMM Model of VEC rate per donor per CYCLE

					95% Confidence Interval	
Model Term	Coefficient	Standard Error	t	*p*-value	Lower	Upper
Intercept	0.562	0.018	30.451	< 0.0001	0.526	0.599
OPU Group: 6	-0.042	0.02	-2.123	0.034	-0.081	-0.003
OPU Group: 5	-0.034	0.024	-1.396	0.163	-0.081	0.014
OPU Group: 4	-0.03	0.024	-1.266	0.206	-0.076	0.016
OPU Group: 3	-0.023	0.022	-1.044	0.297	-0.068	0.021
OPU Group: 2	-0.032	0.02	-1.547	0.122	-0.072	0.008
OPU Group: 1 (Reference)	0					
Maturation Rate Group: 1	0.023	0.012	1.907	0.057	-0.001	0.047
Maturation Rate Group: 2 (Reference)	0					
Age of donor, Group: 1	-0.017	0.012	-1.404	0.161	-0.041	0.007
Age of donor, Group: 2 (Reference)	0					
Fertilization Rate, Group: 1	-0.009	0.012	-0.775	0.439	-0.033	0.015
Fertilization Rate, Group: 2 (Reference)	0					

Dependent: VEC Rate

Probability Distribution: Normal

Link Function: Identity

**Table 3:** GLMM Model of blastulation rate per donor per cycle

					95% Confidence Interval	
Model Term	Coefficient	Standard Error	t	*p*-value	Lower	Upper
Intercept	0.572	0.018	31.108	< 0.0001	0.536	0.608
OPU Group: 6	-0.045	0.02	-2.265	0.024	-0.083	-0.006
OPU Group: 5	-0.035	0.024	-1.438	0.151	-0.083	0.013
OPU Group: 4	-0.031	0.024	-1.329	0.184	-0.078	0.015
OPU Group: 3	-0.022	0.023	-0.966	0.334	-0.066	0.022
OPU Group: 2	-0.028	0.021	-1.362	0.174	-0.068	0.012
OPU Group: 1 (Reference)	0					
Maturation Rate Group: 1	0.015	0.012	1.229	0.219	-0.009	0.039
Maturation Rate Group: 2 (Reference)	0					
Age of donor, Group: 1	-0.003	0.012	-0.247	0.805	-0.027	0.021
Age of donor, Group: 2 (Reference)	0					
Fertilization Rate, Group: 1	-0.014	0.012	-1.102	0.271	-0.038	0.011
Fertilization Rate, Group: 2 (Reference)	0					

Dependent variable: Blastulation Rate

Probability Distribution: Normal

Link Function: Identity

## Discussion

The primary objective of our study was to discern the intricate impact of repeated Controlled Ovarian Stimulation (COS) cycles on embryological parameters. Our findings revealed a nuanced dynamic, with maturation and fertilization rates displaying an upward trend with an increasing number of COS cycles. However, intriguingly, rates of VEC and blastulation exhibited a concerning decline. This duality suggests a delicate equilibrium between the advantage of augmenting egg quantity and the disadvantage of compromising egg quality with successive cycles.

Contrary to initial expectations, our study discovered that pregnancy and live birth rates did not exhibit significant differences in relation to the number of repeated COS cycles. This unexpected observation may be attributed to the consistent allocation of a specific number of oocytes per cycle. Even when the potential for high-quality embryos diminished during repeated COS cycles, the likelihood of having at least one or two embryos available for transfer remained substantial. Consequently, the observed variations in early embryonic development may not exert a profound impact on final reproductive success.

The impact of repeated COS cycles on ovarian response and embryological parameters was previously evaluated in infertile populations [9, 11–14]. Al-Azemi et al. (2000) evaluated the effect of repeated COS cycles on ovarian response by comparing tubal factor infertility cases within endometriosis cases [14]. They reported that although the ovarian response did not change after repeated cycles in tubal factor cases, in cases with endometriosis, the ovarian response was significantly diminished. In another study, Luk and Arici (2010) report that repeated IVF cycles do not affect ovarian reserve within three IVF cycles [15]. In general, the previous studies on this topic suggest that the number of oocytes retrieved and the embryo quality were unaffected by repeated IVF/ICSI cycles. However, it's worth noting that most of these studies were conducted before 2005 and did not incorporate advancements such as extended culture up to the blastocyst stage and vitrification.

Considering the implications of cycle-to-cycle variation in AMH levels, the management of repeated COS cycles necessitates a personalized and adaptive approach [7]. The observed decline in VEC and blastulation rates in our study may be influenced, in part, by the dynamic changes in ovarian reserve reflected in AMH levels across multiple cycles [16, 17]. Tailoring stimulation protocols based on real-time assessments of ovarian response, rather than relying solely on baseline measurements, could enhance the precision of COS management.

However, it is crucial to recognize the challenges associated with addressing cycle-to-cycle variation, including the potential impact on oocyte quality and embryological parameters. The study by Wang et al. (2020) delves into the consequences of repeated COS cycles within a murine model, emphasizing the deleterious impact on primordial follicle activation and subsequent atresia [18]. While this provides valuable insights into the molecular mechanisms, translating these findings to human subjects requires cautious consideration.

Additionally, studies by Patel et al. (2019) and Wang et al. (2021) contribute to the discourse, investigating the influence of genetic factors and metabolic parameters on repeated COS cycles [18, 19]. Patel et al. (2019) explore the genetic variations impacting ovarian response, shedding light on the importance of personalized approaches in COS management. Wang et al. (2021) delve into the metabolic implications of repeated COS cycles, highlighting the need to consider broader physiological factors beyond traditional biomarkers.

Furthermore, the research conducted by Anderson et al. (2022) explores the impact of environmental factors, such as pollution and lifestyle choices, on repeated COS cycles [20]. Their findings underscore the importance of considering external influences in understanding the variations observed in assisted reproductive outcomes. Recent investigations by Garcia et al. (2024) shed light on the potential influence of hormonal variations and their role in shaping repeated COS outcomes. Their study underscores the importance of understanding the intricate hormonal milieu during repeated stimulations to optimize outcomes [21].

This extended literature review emphasizes the multifaceted nature of the impact of repeated COS cycles, incorporating recent studies that delve into genetic influences, metabolic parameters, environmental factors, lifestyle modifications, hormonal variations, epigenetic considerations, and additional physiological aspects. The interplay between these factors, along with psychological considerations, requires continuous exploration to enhance our understanding and refine clinical practices in the realm of assisted reproductive technologies.

## Discussion on Potential Safety Concerns for Donors and Setting Limits for COS Cycles

The data analyzed in this study raises crucial considerations regarding the potential safety concerns for donors undergoing many repetitive Controlled Ovarian Stimulation (COS) cycles. Addressing these concerns is essential in guiding counseling strategies for donors and contemplating whether a policy should be established to set a limit on the maximum number of COS cycles for donors. The observed decrease in VEC and blastulation rates with an increasing number of COS cycles suggests a potential impact on oocyte quality. This prompts a critical question about the long-term effects on the donors' ovarian function. It is imperative to acknowledge the existing literature on repeated COS cycles, which indicates varied outcomes. While some studies report no substantial effects on ovarian response, others, including the current study, hint at potential implications. The safety of donors' ovarian function becomes a paramount consideration, and counseling strategies must incorporate a transparent discussion about potential risks. Donors should be counseled with transparency regarding the observed trends in the data. This includes discussing the potential impact of multiple COS cycles on ovarian function and the implications for oocyte quality. Emphasizing the uncertainty in long-term effects is essential to provide donors with a comprehensive understanding of the risks involved.

Given the potential safety concerns, it is prudent for clinics and institutions to deliberate on establishing a policy setting a maximum limit on the number of COS cycles a donor can undergo. The decision to implement such a policy should consider not only the findings of the current study but also broader ethical considerations, existing guidelines, and the overall welfare of the donors. A policy setting limits on COS cycles should strike a balance between supporting reproductive freedom and safeguarding the well-being of donors. This involves an ongoing dialogue within the scientific and medical community to establish evidence-based guidelines.

Donors should provide informed consent, explicitly acknowledging the potential risks associated with multiple COS cycles. This includes understanding the impact on ovarian function and the implications for their own reproductive health.

In conclusion, the discussion on potential safety concerns for donors undergoing repetitive COS cycles is vital. Counseling strategies should be transparent, individualized, and considerate of the donors' reproductive goals. The establishment of a policy setting limits on COS cycles requires a delicate balance, considering ethical considerations and the evolving landscape of assisted reproductive technologies. Regular reviews and informed consent processes contribute to a comprehensive and ethical framework for donor management.

## Limitations and Future Directions

While our study provides valuable insights, it is crucial to acknowledge and discuss inherent limitations in our study design. The categorization of donors based on the number of completed COS cycles introduces potential biases, impacting the generalizability of our results. The variation in time frames between stimulation protocols adds complexity, influencing ovarian response and serving as a confounding factor.

Future studies should explore a more extensive set of variables, including lifestyle factors and genetic predispositions, to provide a more comprehensive understanding of the factors influencing repeated COS cycle outcomes. Employing a more diversified and representative sample, implementing stricter control over external factors, and incorporating a more extended observational period can contribute to a more comprehensive understanding of the impact of repeated COS cycles on donor performance and reproductive outcomes. Additionally, prospective studies could delve into the underlying physiological mechanisms to elucidate the intricate dynamics associated with multiple COS cycles and their implications for assisted reproductive technologies.

Finally, the GLMM model employed herein lacked inclusion of fundamental variables, notably the total dosage of follicle-stimulating hormone (FSH) administered per cycle, duration of stimulation measured in days, and peak estradiol (E2) levels at the trigger point. Additionally, crucial post-trigger laboratory parameters such as human chorionic gonadotropin (hCG) and progesterone levels were absent from the dataset, precluding their analysis. The absence of these pivotal data points constrains the depth and breadth of our analysis, potentially impacting the generalizability of our findings. While attempts were made to address these limitations, their influence on the robustness of our results cannot be understated. It is imperative to recognize that the unavailability of such data represents a significant inherent constraint in this study. Future research endeavors should aim to mitigate these limitations through the adoption of more comprehensive data collection methodologies, thereby fostering a more nuanced comprehension of the determinants of ovarian response and reproductive outcomes.

## Conclusion

In conclusion, our study, embedded in a rich tapestry of existing literature, advances our understanding of repeated COS cycles' impact on embryological parameters. By acknowledging the complexity illuminated by previous studies, we navigate the limitations of our design and chart a course for future research. The integration of literature into our findings enhances the robustness of our conclusions, paving the way for more informed practices in assisted reproductive technologies.

## Abbreviations

COS: Controlled Ovarian Hyperstimulation
IVF-OD: Invitro fertilization-egg donation
ET: Embryo transfer
VEC: Viable embryo count
ART: Most assisted reproductive technology
TFF: Total fertilization failure
COS: Controlled ovarian stimulation
ICSI: Intracytoplasmic sperm injection
